# Jumping is not just about height: Biosocial becomings as an integrative approach in understanding contextualized jump performance in Maasai society

**DOI:** 10.1371/journal.pone.0278547

**Published:** 2022-12-01

**Authors:** Xiaojie Tian, Yushi Yanohara, Francis M. Mwangi, Natsuki Sado

**Affiliations:** 1 Faculty of Health and Sport Sciences, University of Tsukuba, Tsukuba, Japan; 2 Japan Society for the Promotion of Science, Tokyo, Japan; 3 The Center for African Area Studies, Kyoto University, Kyoto, Japan; 4 Department of Physical Education, Exercise & Sports Science, Kenyatta University, Nairobi, Kenya; University of Belgrade: Univerzitet u Beogradu, SERBIA

## Abstract

Studies focused on jumping performance in humans have so far investigated either its biological or sociocultural significance, with very little attentions paid to the inseparable relations of these two aspects in daily life of people. Integrating both ethnographic and biomechanical methods, this research investigated the biosocial features of the jump performance of Maasai youth in its most well observed context, the wedding ceremony. Ethnographic data were used to explain the social status of participants, the physical movements and singing tempo of performers, and their interactions. Biomechanical methods were applied to assess the heights and frequencies of identified repetitive double-legged vertical jumps (n = 160, from 15 male youths). All youth performers followed a certain posture pattern, paying specific attention to their final landing. Large variations exist in their jumping heights [coefficient of variation (CV) = 0.237]; however, the frequency in jump repetitions were maintained with the least variations (CV = 0.084). Cheering interactions were confirmed, but with no significant difference in height between the cheered and non-cheered groups. These results indicate that the Maasai youths did not compete for jump height during local ceremonies. Rather, they emphasized the rhythmical retention of jumps, corresponding to other youth mates who were singing alongside. In the broader context of human behaviors, the analysis addresses the diverse meanings of motor performances in different daily contexts that reject the generalized sports regime of “higher/faster-the-better”.

## Introduction

Jumping, one of the most basic human body movements has been studied by scholars in both the biological and sociocultural fields. However, limited collaboration has been undertaken between these two groups. In biological studies, the mechanics of physical movements has been investigated for more than five centuries. Scholars focus on the biological features of the body in jumping movements and investigate the mechanisms of different kinesiological phenomenon such as the tissues, organ and the whole musculoskeletal system, their correlations and interactions during this movement [1–5]. These studies make numerous contributions to a better understanding of the body movements in modern sports, whereas little attention is paid to its various forms in non-sports contexts. Consequently, height is emphasized over other features in jumping. From the late 20th century, the development of body culture studies by sociocultural scholars have questioned the western-centric approach towards human performance by exploring the symbolic, emotional, and sociocultural features of the physical performances of people in culture groups [6, 7]. These studies contribute to a better understanding of the diversity in body movements, however inter-field discourse is very limited. It is worth noting that body performance, as part of daily human experiences, its biological, psychological, and sociocultural features are simultaneously and inseparably intertwined. Comprehensive understanding requires an integrative approach that blurs the boundaries between biological/natural and sociocultural in academic discussions [8–11]. This study focuses on the jump performance of pastoralist Maasai in Southern Kenya. It explores possible collaboration and conversations between the two groups of studies so as to achieve a comprehensive understanding of the biosocial features of the body performance of Maasai.

Since the late 19th century, colonial officers and western adventurers documented the sports-like body performances of pastoralist Maasai, including running, spear throwing, and jumping. The images they produced have been widely utilized in tourism sectors [12, 13]. At the same time, scholars from both biological sciences and cultural studies also explored this topic. Biological studies focused on the body and jumping performance of Maasai and explored both anthropometrical and biomechanical features of individuals [14–16]. They analyzed the foot structures of Maasai in standing and walking, and measured the muscle-tendon functions, the power and force in jumping mechanics, and movement of both the upper and lower body of youth during repetitive jumping. These studies were conducted in a controlled laboratory environment. The biomechanical functions of the movements were compared to existing literature, most of which focused on athletes in different types of modern sports. Studies focused on the height and fitness of individuals as such, often ignoring the intentions, perceptions, and actual daily practice of local people. Many scholars in body culture discussions have probed such “natural approaches” towards body apparencies and performances in non-western societies as the imagination of the outsiders [12, 17]. One good example is Bale’s critical study on the western perceptions towards jump performance of Tutsi in Rwanda and Maasai in Kenya during the colonial era [12, 18, 19]. He analyzed existing images of the jump performance of Tutsi and Maasai that were recorded by western visitors during the late 19th and early 20th century. These early visual images were commonly set in a western sport context, using bars and ropes to measure the height of an individual’s jump [12, 20]. The conceptualization of these athletic images reflected the “native” or “savage” interpretations of local people, following the by-then widely accepted racialism lens. Such “Western models of recording athletic performance at the individual level”, as Bale suggests [18], strongly ignored, or even neglected local attitudes, voices, and ways of doing in presenting and interpreting their own bodies. From the late 20th century, criticism of such modern sports-defined interpretations of body performances drew attention to “body culture”, which emphasizes the diverse meanings of body performances as contextualized practices of people in their daily life in time and space [7]. It is worth noting that as the conversations between scholars in natural and sociocultural studies remains limited, the concept of body culture is rarely referred to in both anthropometric and biomechanical studies.

At least two challenges should be addressed due to this nature and culture segregation in research. First, natural sciences methods continue to be narrowly applied in evaluation of the body within confined modern sport contexts. They are rarely used for discovering the mechanisms of body performances of people within non-sports daily conditions. Recent measuring of the *Gusimbuka-Urukiramende* and the interpretation of its height in terms of its competitiveness at the Olympics [21] presents this first challenge. The above comment does not criticize the biomechanical approach itself in measuring this jump performance. As earlier questioned by Bale, photographs taken during the colonial era “makes the event a European production” [18], which neglect the local ways of doing and interpretations of body performance. For the practitioners in the recorded *Gusimbuka-Urukiramende*, the excellence of this jump may not lie in its height; nor may they have been interested in raising individual jump heights in daily life. The problem is that existing representations have contributed to the fragmented understanding of *Gusimbuka-Urukiramende*, with more concern given to the high jump in an “imagined” sports world [18, 22]. The second challenge relates to the first, but in a more practical and complex way due to the recent dissemination of local body as “tradition” in different sectors. Taking the Maasai jump for instance, the exhibitions of the “dancing of Maasai warriors” in tourism cites [23], and the interpretation of “traditional warrior skills” in wildlife conservation programs such as the Maasai Olympics [13] reflect this challenge. In both cases, notwithstanding the participation of local people, catering to the body imaginations of people from other cultures [24] were considered the only solution to gain external attention. As such, to showcase the “nature” of Maasai and their “traditional body skills” induces the continued misinterpretations of Maasai body culture in the concurrent development discourse [22].

To deal with the above challenges, more attention should be given to local ways of doing, moving beyond the idealized “natural” and “cultural”, and the “body” and “mind” dichotomy [8, 25, 26]. From the late 20th century, scholars from various disciplines noted the correlation between natural and cultural in human behaviors and development and suggested new approaches towards an integrative understanding of this topic. For instance, in biomedical fields, the biopsychosocial model [9] is widely applied in both academic discussions and treatment practices [27, 28]. This model takes a holistic approach towards an individual’s health problems by considering the correlations and mutual influence among biological, psychological, social, cultural, and spiritual factors in life. In this model, the links between multiple aspects were emphasized, and these features were considered independent variables. In anthropological studies, the integration is approached with more attention given to the intertwined and ongoing co-developing features of body experiences under the term biosocial [10, 25, 29, 30]. Research in this field refuses to consider humans “as discrete and preformed entities but as trajectories of movement and growth”, in a state of continuing “becomings, (which) are brought forth within a field that is intrinsically social and biological” [31]. To capture the body movements from the perspective of biosocial becomings, we may situate the biomechanical features―for instance the height, speed, and postures of performers―back to the daily life of people, and re-consider their significance as a part of body techniques and habitus in time and space [32, 33]. Integrating the ethnographic approach with biomechanical methods, we are able to re-value the mechanisms of this the jump performance of Maasai youth as contextualized body culture. The features of the jumping height, frequency, postures, and interactions throughout the performance in both individual and interpersonal levels could be further explored, firstly, by considering the implicit social norms towards personhood, particularly manhood; and secondly, of its relation to the implicit bodily habits, dispositions and interactions in time and space. To shed light on these aspects, the following section briefly explains the life of Maasai and local lexical terms in relation to jump behaviors.

The Maasai live across the arid and semi-arid land in southern Kenya and northern Tanzania. Being generally known as pastoralists, livestock play important roles in the daily life of Maasai having both sociocultural and subsistence significance [34]. While maintaining seasonal livestock grazing, local people have long adopted other forms of subsistence, such as farming and wage labor in cities to cope with changes [35]. The development of the jumping performance of “warriors” in tourism is also one such example of local adaptions [23]. The so-called “Maasai warriors” are male youth (*ilmurran* in the local Maa language), who graduate from boyhood by forming their own age-set following the local age system. This youthhood continues for about 10 to 15 years, during which the youth take on the social responsibility of long-distance herding and safeguarding both livestock and people. In daily life, *ilmurran* express their social identity by long hair, colorful clothes, bead ornaments, and a walking stick. The stick is used as an extension of the body during communication, such as pointing in a formal meeting to give one’s opinions [36]. In daily life, Maasai use different terms to describe jump practices [37, 38]. The hypernym term *aipid*, to jump, generally indicates all different forms of jumping movements. Under it, the verb *aigis* refers to the all forms of double-legged jumps, either for distance or height. Conversely, the verb term *aid* refers to all forms of vertical jumps that cross over something. It is similar to the verb *alang*, which means to step up and jump across. Another verb *aitiam* means to hop from one place to another, either on one leg or both legs. The verb *adumu* refers to the jump movements in dancing. This term is also used as a noun to indicate the vertical high jump dance of the *ilmurran*. This dance is practiced by the youths as part of their play (*enkiguran*). Many other pastoralist societies of the same Nilotic language group also have similar age and gender restricted body performance [39].

This study focuses on the *adumu* of Maasai youth in a wedding ceremony. The wedding ceremony is one of the most important and celebratory life events that *ilmurran* experience during the latter stage of youthhood. This life event signifies a paramount step for these young men, to build their own family as they move forward into elderhood. *Adumu*, as it represents the youth group, is indispensable in this life event.

## Methods

### Data collection

Data were collected by the first author through long-term fieldwork in a Maasai village in Southern Kenya from November 2019 to January 2020. After explaining the purpose of this research, the author was invited by the youths to attend local wedding ceremonies to observe their *adumu*. The *adumu* was recorded in one wedding ceremony using a video camera (FDR-X3000, Sony; resolution: 1920x1080, frame rate: 60p). After the video recording, the sociodemographic data of the performers, including their age and subsistence forms were confirmed with the help of a local research assistant. From this video data, a 7-minute clip which had captured the singing and whole-body movements of all male youth performers (n = 17) were selected for further analysis.

**Fig 1:**
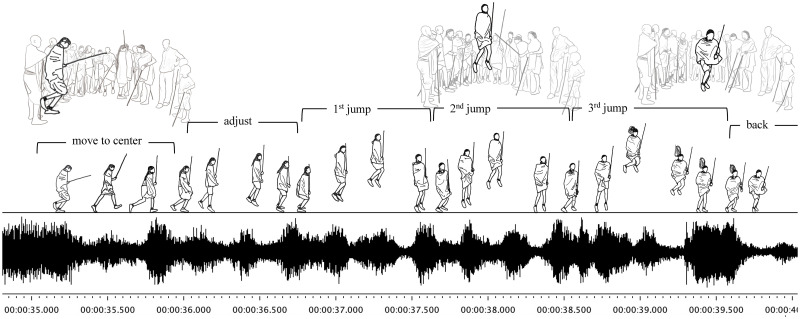
Illustration of one jump session following the rhythm.

The *adumu* began with all participants standing in a half circle and singing different chant lines. The chants were continued throughout the *adumu* serving as its base rhythm. In-between the chants, soloists sang lyrics one-by-one, during which each youth moved into the center of the half circle to perform double-leg jumps (DLJ). In details, when the singer starts to sing, each jumper initially moved into the center of the group (Fig 1). At this stage, the jumper adjusts his steps carefully through walking and hopping to match his movement with the rhythm. When the jumper is ready, he then bends his knees and lower the upper body to start continuous DLJ. The DLJ as the main performance is repeated for several times till the final landing. Throughout the DLJ repetition, the jumper keeps the upper body straight and minimized whole body movements. The postures of both legs are flexible―either close or slightly open during upward progression. The arms are intentionally kept steady and motionless, generally with one hand holding a stick and the other holding the clothes. This posture is kept throughout the apex of jumping and landing during the DLJ repetitions. The performers usually emphasize their final landing of the last DLJ in a jump session by flexing the knees towards the upper body. This flexion sometimes accompanies the bending of both the head and upper body downward facing the ground, which would make the jumper’s long hair float up towards the sky. Following this movement, the jumper lands with a loud sound. Upon landing, the jumper then quietly and quickly move from the center position and return to stand with the group. In this progress, we are able to conduct data analysis by focusing on the tempo of the raw singing audio and the biosocial features of body movements on both individual and inter-personal levels.

### Data analysis

Firstly, the general flow of movements, including jumping, non-jumping body movements of individual performers, and the cheering interactions among performers in the group were visualized using the ELAN behavior analysis software (ver. 6.2). Secondly, the multi rhythms, that is the repeated patterns of the chants and solo singing (4 youths, 6 times) were transcribed using the Studio One (version 5.5.2.86528) audio software by the second author. Following the transcription, the main tempo (i.e., the beats per minute) of the rhythms throughout the 7 minutes recording were further analyzed using the Melodyne Studio (version 5.1.1.003) audio software. From video data, the number of DLJ from each jumper were identified and coded. Separately, the toe-off instant (0.000s) and ground contact instant of each DLJ were coded using Final Cut Pro by the first author and a research assistant who is not familiar with this project. From this data, the flight time, *t*, of each DLJ (i.e., from toe-off to landing), and the contact time from toe-off to the next toe-off in DLJ repetitions was calculated. We also defined the sum of contact time and flight time between two toe-offs as time set of one DLJ, or the rhythm of jumping in this study. As the DLJ was repetitively continued, this time set variable can be used to examine the jumping frequency during the recorded wedding *adumu*. Also, assuming that the BCOM height at the instant of toe-off and of landing is equal and that the air resistance is negligible, BCOM motion behaves as a vertical throw-up motion (i.e., only having the gravitational acceleration). The achieved BCOM height, *h*, can be calculated from the law of conservation of mechanical energy:

h=v022g (1)

where *v*_0_ is the vertical velocity at the instant of toe-off, *g* is the gravitational acceleration. As the instant of highest BCOM (t2) means the instant at zero vertical velocity:

v0−g(t2)=0 (2)

Substituting Eq 2 into Eq 1 yields:

h=(gt2)8 (3)

From all the identified DLJ, we calculated 157 BCOM heights utilizing Eq 3. This equation has been confirmed as reliable and it is widely applied by coaches and physical educators to assess the jumping performance of athletes and students [40, 41].

As the Jump height and time parameters that were coded by two observers, an inter-observer agreement was established. The reliability in the jump height and time parameters was confirmed by using the intraclass correlation coefficient (ICC) in RStudio (irr. package). Following Cicchetti’s (2001) four categories of agreement levels (i.e., < 0.40 poor, 0.40–0.59 fair, 0.60–0.74 good, and 0.75–1 excellent), excellent reliability was confirmed (ICC > 0.88, 95% CI). On the interpersonal level, the timing, methods, and the types of cheering interactions were extracted from the video data. We also identified the targeted person of the cheering actions to assess the types or the reasons for cheering. For instance, if one youth raised his stick to tap the stick of the singer, this cheering interaction is categorized as being towards the singer. The DLJ height was compared between the cheered and non-cheered groups through an independent sample t-test using open-source software Jamovi (version 1.6.23.0). Together with the descriptions of the general body movements flow in *adumu*, these data are used to explain the implicit bodily habits of the practitioners.

This study was approved by the ethical advisory committee of the University of Tsukuba (IRB ID: 30–109) and was guaranteed with official research permission from the National Commission for Science, Technology and Innovation (NACOSTI) in Kenya (Projects No. NACOSTI/P/19/85899/28060 and No. NACOSTI/P/19/17728/29922). For both data collection, informed consent was obtained from all the youth participants, and assent was obtained from the host family members at the wedding ceremony and their guests.

## Results

In the 7 minutes video, four soloists sang six songs, all of which shared a similar melody and followed the rhythm of the chanting. The main tempo of the singing ranged from 137 BPM to 139 BPM (i.e., 2.28–2.32 Hz). In total, 17 youths (*ilmurran*) participated in the *adumu* (Table 1). One youth (Ke) left the group soon after the dance started. Another youth (KL) did not jump as he lacks experience in *adumu*. The remaining 15 youths performed 50 jumping sessions, of which, 1 to 34 times of DLJ per person were performed. In general, for both jumper and soloist, there are no specific rules to determine the times of individual performances. All the youths are free to join or leave at any time. Two youth visitors from another village joined the *adumu* in the middle (Jn and Nt) and performed jumping and singing. Other community members were allowed to watch nearby, even standing in the same group. In the video, a male elder accompanied by several boys stood at the edge of the group. The elder quietly watched the performance. The boys mimicked the youth jumping by watching and following the rhythms of the singing. Adult women had also walked by the group, but none stopped. The interactions for cheering were only conducted among the youth themselves, who cheered with each other by cross tapping their sticks. Many youth participants who were herders dressed in traditional style clothing, including the loosen colorful blanket clothes, sandals made from car tires, and had worn bead ornaments on their heads, necks, belts, legs, and arms. While jumping these ornaments create sounds, which enrich the rhythms of their performance.

**Table 1:** Sociodemographic features and performance patterns of individual youth.

Participant	Age	Career	Singing	No. of Jump Sessions	No. of Identified DLJ
Ke	28	herder	chants	-	-
KL	25	town worker	chants	-	-
Ly	26	herder	chants	2	1
Le	29	herder	chants	1	1
Nt (visitor)	n.d.	herder	chants	1	2
Sp	27	herder	chants	2	4
Sn	25	herder	solo + chants	1	5
Np	31	herder	chants	1	6
Mn	27	herder	solo + chants	4	7
Jn (visitor)	n.d.	herder	chants	2	7
Kw	30	game scout	chants	3	8
NI	26	herder	solo + chants	3	8
SI	24	herder	chants	4	11
Nk	25	univ. student	solo + chants	4	12
Lk	28	herder	chants	8	21
Mb	27	herder	chants	6	33
Kt	27	herder	chants	8	34
Total				50	160

**Fig 2:**
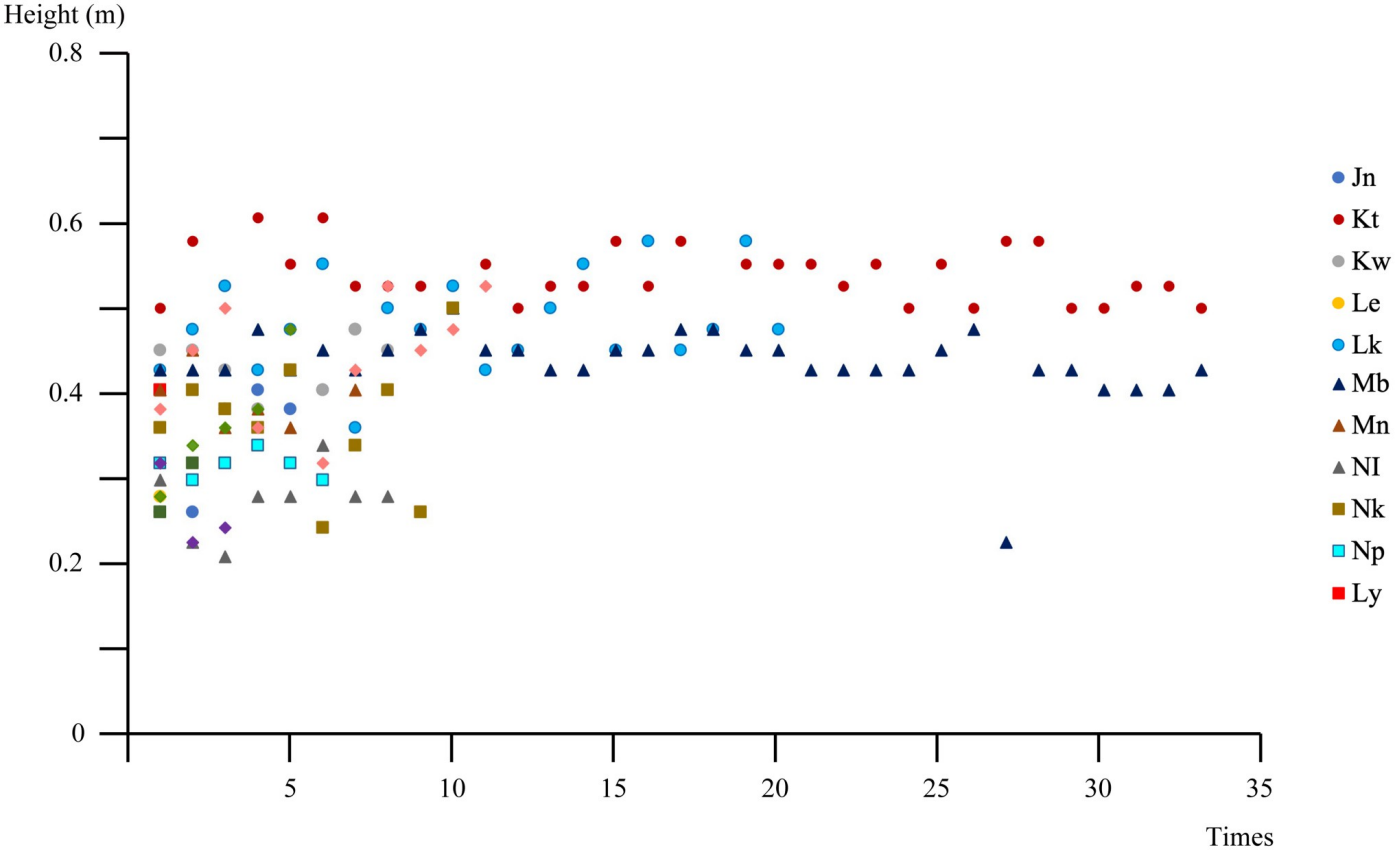
Distribution of BCOM heights for each jumper. (n = 155).

From the total of 157 double-leg jumps, the mean time set for these DLJ has the least variation in contrast to other variables (Table 2). In comparison, the BCOM heights of identified DLJ show greater variations. This means, all the jumpers retained normal velocity during the DLJ repetitions, notwithstanding the height reached. Among the individual jumpers, the BCOM heights of their DLJ have significant differences, ranging from 0.229m to 0.608m. Moreover, the youths, whose DLJ comprised higher mean BCOM heights, tended to perform more jumping sessions during the *adumu* (Fig 2). To further convert the DLJ time set into jump frequency, the derived mean (± SD) is 1.228 (± 0.298) Hz. This frequency is likely to correspond to the main tempo (2.28–2.32 Hz), but more detailed investigations are still needed.

**Table 2:** Time and height features of coded DLJ. (n = 157).

	First Observer			Second Observer			ICC	Effective Sample Size (times)
	Mean (SD)	CV	95% CI	Mean (SD)	CV	95% CI		
Contact (s)	0.251 (0.059)	0.234	0.241~ 0.260	0.229 (0.047)	0.207	0.221 ~ 0.237	0.905	153
Flight (s)	0.586 (0.076)	0.129	0.574 ~ 0.598	0.609 (0.062)	0.102	0.599 ~ 0.619	0.943	155
Set (s)	0.829 (0.070)	0.084	0.819 ~ 0.840	0.830 (0.071)	0.085	0.819 ~ 0.841	0.892	156
Height (m)	0.428 (0.101)	0.237	0.412 ~ 0.444	0.460 (0.090)	0.196	0.446 ~ 0.474	0.941	155

^a^SD: standard deviation; CV: coefficient of variation; CI: confidence interval; ICC: intraclass correlation coefficients.

^b^Abbreviation indicates the unit of measurements, s: second; m: meter.

Large variations in BCOM height could also be observed within the DLJ of individuals. Table 3 shows the features of BCOM heights of 13 youths, who had performed DLJ more than twice. Although the youth who performed more DLJ have greater BCOM heights, large variations in BCOM heights exist in both higher and lower groups. Together with our other findings, these results suggest that the attention of the youth performers were more likely focused on rhythm retention throughout the DLJ repetitions. As such, jump movements which could have been higher, were sufficiently controlled so that the performer could land on time to maintain the rhythm of the group. This can be further confirmed from the cheering practices in the latter part.

**Table 3:** BCOM height differences in the DLJ of individuals.

Participant	DLJ times	Mean (m^a^)	SD^a^ (m)	CV^a^ (m)
Nt	2	0.293	0.029	0.098
Sp	3	0.237	0.060	0.254
Sn	5	0.370	0.064	0.172
Np	6	0.318	0.014	0.043
Mn	7	0.388	0.035	0.089
Jn	7	0.370	0.073	0.197
NI	8	0.277	0.038	0.137
Kw	8	0.436	0.028	0.064
Nk	10	0.336	0.103	0.307
SI	11	0.447	0.064	0.143
Lk	20	0.484	0.051	0.106
Mb	33	0.437	0.043	0.099
Kt	34	0.526	0.079	0.150

^a^m: meter; SD: standard deviation; CV: coefficient of variation.

During the *adumu*, the cheering occurred either in-between or during the jumping performance (Table 4). From the total of 50 sessions of jumping, cheering interactions were practiced in 28 sessions. The timing for cheering was quite flexible, most being conducted either before a jump session or after the last landing in a jump session when the jumper walked back to the half-circle. We identified three types of cheering behavior: for jumping performance, for singing, and for friendship, of which the first two types were more frequently practiced.

**Table 4:** Features of celebrations in *adumu*.

Identified Features in Categories		No. of Sessions	Proportion^a^
**Timing**	during performance	6	0.21
	in-between performance	22	0.79
**Types of cheering**	to friends	2	0.07
	towards singer	12	0.43
	towards jumper	14	0.50

^a^Proportion of each category among the 50 sessions.

Among the youth who jumped, seven were cheered by others for their performances (Table 5). Comparing the BCOM heights of DLJ of the same individual when being cheered and not being cheered, we found that first, there is no significant difference between the non-cheering and cheered DLJ in BCOM heights. Secondly, the BCOM height of the cheered group did not go higher than the non-cheered. This feature is common in all the six focused youth jumpers. In terms of the dispersions, except Mn, the BCOM height of DLJ in the cheered groups were less dispersed than the non-cheered groups.

**Table 5:** Height difference of individual’s jumps in celebrative and non-celebrative conditions.

Participant^a^	Cheering for jump	No. of DLJ (%)	BCOM Height Mean (SD^b^)	P-value^c^
Mb	Cheered	8 (0.20)	0.457 (0.027)	0.123
	Non-cheered	33 (0.80)	0.457 (0.044)	
Lk	Cheered	12 (0.60)	0.494 (0.053)	0.245
	Non-cheered	8 (0.40)	0.477 (0.061)	
Kw	Cheered	4 (0.50)	0.442 (0.030)	0.310
	Non-cheered	4 (0.50)	0.430 (0.032)	
Kt	Cheered	20 (0.61)	0.528 (0.103)	0.418
	Non-cheered	14 (0.39)	0.522 (0.231)	
SI	Cheered	3 (0.27)	0.447 (0.059)	0.504
	Non-cheered	8 (0.73)	0.447 (0.073)	
Mn	Cheered	4 (0.57)	0.391 (0.050)	0.591
	Non-cheered	3 (0.43)	0.400 (0.047)	

^a^Np, who performed DLJ 6 times in his single jump session. He was cheered for his jumps, but as this action is not comparable, his DLJs are not listed in this table.

^b^SD: standard deviation

^c^Independent samples t-test (Hₐ: Cheered > No-Cheer, p<0.05)

## Discussion

Jumping as one of the most basic movements in humans is practiced in various forms as contextualized. Understanding how people jump in non-sports contexts contribute to a better understanding of the significance of body movements considering both human nature and culture. This study examined the contextualized meanings of the well-known jump performance, the *adumu* of Maasai youths from the perspective of biosocial becomings. We found large variations exist in the jumping heights of youth. The frequencies in jump repetitions were maintained with the least variations compared to other variables, i.e., the flight time, landing time, and jumping height. Moreover, cheering interactions were confirmed targeting not only the jumpers, but also the soloists, and friends. For the jumpers who were cheered, no significant difference in heights between the cheered and non-cheered jumps. The evidence shows that the Maasai youth did not compete for jump height in *adumu* during the wedding ceremony.

Following these findings, we discuss the biosocial features of *adumu* in both individual and interpersonal levels, and the sociocultural norms that afford this performance. First, we highlight the responsiveness of this body performance. In presenting oneself for jumping, an individual jumper first follows the tempo of singing and the implicit performance patterns of moving in, jumping, and moving back. This individual showcase emphasizes the jumping postures in keeping the upper body straight and vertical during jumping, and the intentioned rhythmic landing, which, is in response to the singing, moving the performance of the group towards the climax. The cheering with sticks for both jumping and singing also reflect the highly regarded responsiveness of individuals towards other group members through body movements in *adumu*. Secondly, we stress that the jumping and singing are valued together as a playful process in *adumu*. The casual joining in and leaving of youth members and the slight differences between the cheering for jumpers and singers reflect this feature. These findings indicate that perceiving *adumu* as merely a sports-like competitions is biased. It over emphasized some aspects of the bodies in *adumu* and failed to comprehensively capture the dynamic and adaptive embodied features of the movements and interactions of individuals during the *adumu* in time and space. Giving emphasis to the co-making of rhythmics, we value *adumu* as a form of dance that is rich with playful and interactive elements, through which the youth performers unite.

Going back to the aforementioned two challenges in the existing literature, the jumping performance in *adumu* of Maasai differs considerably from jump competitions in western sport contexts [18]. For individuals, jumping higher was not the goal of this performance. This could be confirmed from various perspectives throughout the performance. For instance, the youths gain equal opportunities to present themselves and interact with others through both jumping and singing, notwithstanding the height reached (mean height 0.428 ± 0.101m for the 15 jumpers). Those who did not jump high were also cheered for their performances. It is worth considering what was identified in the mechanics of individual jumps in previous studies under laboratory conditions [15, 16] actually meant to Maasai in relation to their daily body habitus. It is also interesting to further investigate and compare the mechanics of different jumping experiences of Maasai youth in their daily life, for instance jumping in schools or the jump competitions in the Maasai Olympics [13].

Secondly, the interpersonal and communicative features of both jumping and singing in *adumu* are highlighted in the ways how the youths develop the embodied cooperation throughout the *adumu*. The continued chanting of group members at a retained speed afforded the rhythmic jumping of individuals. We may conclude that embodied socialization and the musicality [42] are the two main characteristics of the jump performance of Maasai youth worth further exploration. As we selected data considering the continuation of *adumu*, no restarts or pauses were observed. However, as observed by Spencer [39] on the youth dance of another Maa speaking pastoralist group Samburu, a restart in dancing would occur for instance, due to the disruptions that induce the collapse of group chanting or the lack of proper response from the others to the singing of the soloist. From this perspective, the rhythmic jumping of individuals is captured as part of the ongoing musicking [43] of the youth group. This perspective of musicking also helps us to better explain why the Maasai youths who on average jumped higher performed more jump repetitions. If we assess these youths who jump higher and with greater repetition as having higher physical fitness, their physical ability may reflect the correspondence of the jumps with the singing. This is to say that the physical fitness also has great interpersonal and social significance which is worth further exploration. After all, further investigation requires greater attention to the process of synchronization in the rhythmic body movements and singing, which could improve current understandings of the social identity making of the youths in current Maasai society.

Another point we intend to address is also related to the social identities of youth in Maasai society. Age system and gender roles are important social organizations of the Nilotic language groups [44]. Such gender-age roles are not stable but always in transition so as to cope with external changes [45]. This can be also observed in the jumping performance of youth. In daily life, the youths participate in various forms of dispersed labor. The jumping and singing provide them the opportunity to communicate regardless of their regions, clans, economic status, or educational level. The youths enhance their sense of being in the same age-set through moving and singing together in a collaborative manner. This again, is completely different from the competitive jumping practices in a modern sports context. In terms of jumping skill transmission, during the *adumu*, when the elders and children participated as spectators, the children gained the chance to mimic the youths at a very close distance. In this way, children informally and embodied learn how to jump while developing their knowledge of social relations and the institutionalized age groups. To better understand the (dis-)continuations of this practice as a Maasai tradition of over time, more attention should be drawn towards the jumping practices of children and the youthhood *adumu* memories of the elders.

Lastly, several limitations exist in the current study. First, the work focused only on the body performances of *adumu* at a wedding ceremony. Although the *adumu* was frequently observed in this life event, these findings should not be generalized to represent the various jump experiences of Maasai in other daily social occasions. But this study could serve as the first step towards the comprehensive understanding of the various types of jumping performances of Maasai youth and other cultural groups in time and space. Furthermore, as the current study only focused on body movements, verbal descriptions of local youths towards their own performances were not considered. Further investigations should explore local voices, which could be combined with findings from this study to examine the *intentions* of practitioners, which are one of the most important variables that engender the jump and singing behaviors during *adumu*. Notwithstanding the limitations, the findings of this study highlight the effectiveness of cross-fields methods applications for moving beyond the natural-cultural dichotomy in current motor movement discussions.

## Conclusion

The physical activity or the motor movements of human beings represent a rich variety of forms in daily practice, which are shaped by and in turn, creatively enrich local life and culture. Findings from this study highlights that the significance of jump performance in Maasai society addresses the togetherness of the male youth in the same age group, through the co-making and sharing of the body tempo in both jumping and singing. As such, the height reached by an individual gained less attention in comparison to the strategies that an individual creatively developed for moving together with others. This study emphasizes such rich means of human locomotion that go beyond modern sports contexts. Moreover, such rich forms of kinematics as body culture are not limited to Maasai youth but could be generally observed in many other non-industrialized societies, which should be the focus of future work.

## Supporting information

S1 Data (XLSX)
